# Liver Cancer-Specific Isoform of Serine Protease Inhibitor Kazal for the Detection of Hepatocellular Carcinoma: Results from a Pilot Study in Patients with Dysmetabolic Liver Disease

**DOI:** 10.3390/curroncol29080431

**Published:** 2022-07-31

**Authors:** Gian Paolo Caviglia, Aurora Nicolosi, Maria Lorena Abate, Patrizia Carucci, Chiara Rosso, Emanuela Rolle, Angelo Armandi, Serena Aneli, Antonella Olivero, Alessandra Risso, Davide Giuseppe Ribaldone, Christian Fermer, Giorgio Maria Saracco, Silvia Gaia, Elisabetta Bugianesi

**Affiliations:** 1 Department of Medical Sciences, University of Turin, 10126 Torino, Italy; aurora.nicolosi@unito.it (A.N.); marialorena.abate@unito.it (M.L.A.); chiara.rosso@unito.it (C.R.); angelo.armandi@unito.it (A.A.); antonella.olivero@unito.it (A.O.); davidegiuseppe.ribaldone@unito.it (D.G.R.); giorgiomaria.saracco@unito.it (G.M.S.); elisabetta.bugainesi@unito.it (E.B.); 2 Gastroenterology Unit, A.O.U. Città della Salute e della Scienza, 10126 Torino, Italy; pcarucci@cittadellasalute.to.it (P.C.); erolle@cittadellasalute.to.it (E.R.); alrisso@cittadellasalute.to.it (A.R.); sgaia2@cittadellasalute.to.it (S.G.); 3 Department of Sciences of Public Health and Pediatrics, University of Turin, 10126 Torino, Italy; serena.aneli@unito.it; 4 Fujirebio Diagnostic AB, 41458 Gothenburg, Sweden; christian.fermer@fdab.com

**Keywords:** diagnostic accuracy, HCC, LC-SPIK, NAFLD, PIVKA-II

## Abstract

Reliable non-invasive biomarkers for the surveillance of patients at risk of hepatocellular carcinoma (HCC) development represent an unmet medical need. Recently, the liver-cancer-specific isoform of serine protease inhibitor Kazal (LC-SPIK) has been proposed as a valuable biomarker for the detection of HCC in patients with chronic liver disease of viral etiology. In the present study, we assessed the diagnostic accuracy of LC-SPIK, alone or in combination with standard serologic biomarkers (i.e., alpha-fetoprotein and protein induced by vitamin K absence or antagonist-II, PIVKA-II), for the detection of HCC among patients with dysmetabolic liver disease. A total of 120 patients with non-alcoholic fatty liver disease (NAFLD), including 62 patients with a diagnosis of HCC and 58 with cirrhosis but without tumor, were retrospectively analyzed. The serum levels of LC-SPIK were measured by enzyme-linked immunosorbent assay (ImCare Biotech, Doylestown, PA). The serum LC-SPIK values were significantly different between patients with HCC (24.3, 17.6–39.8 ng/mL) and those with cirrhosis but without tumor (11.7, 8.7–18.2 ng/mL) (*p* < 0.001). By receiver operating characteristic curve analysis, we observed an area under the curve (AUC) of 0.841 for the detection of HCC; the combination with PIVKA-II further increased the accuracy to AUC = 0.926 (cross-validation). The promising results observed in the present pilot study foster additional research to investigate the usefulness of LC-SPIK for the stratification of the risk of HCC development in patients with NAFLD and advanced liver disease.

## 1. Introduction

Hepatocellular carcinoma (HCC) is the most common form of primary liver cancer, with 905,677 new cases and 830,180 deaths registered in 2020 worldwide [1]. Irrespective of the underlying liver disease etiology, liver cirrhosis represents the principal risk factor for HCC development [2]. Therefore, patients with cirrhosis should undergo surveillance programs in order to detect HCC at earlier stages, thus allowing potential curative treatments [3].

According to international guidelines [4,5], the surveillance of patients at risk of HCC development should rely on ultrasound (US) examination performed every six months, while the usefulness of circulating biomarkers is still largely debated. However, US is an operator-dependent method with suboptimal efficacy for the discrimination between regenerative or dysplastic nodules, and HCC in a cirrhotic liver; furthermore, US sensitivity for small HCC nodules is poor [6]. Conversely, serum biomarkers, whose measurement is operator independent, could represent a valuable diagnostic complement in the setting of HCC surveillance. In addition, the scenario of medical treatment in HCC is rapidly changing [7,8]; given the recent availability of novel tyrosine kinase inhibitors and biological drugs for the treatment of advanced stage HCC, biomarkers for treatment allocation and outcome prediction represent an unmet need.

The liver-cancer-specific isoform of serine protease inhibitor Kazal (LC-SPIK) is a specific SPIK isoform exclusively secreted by liver cancer cells, able to inhibit serine protease-dependent cell apoptosis induced by cytotoxic T lymphocytes and natural killer cells [9]. In a recent study including patients with chronic liver disease of viral etiology, LC-SPIK showed remarkable diagnostic accuracy for the identification of patients with HCC [10].

Given the promising results observed in patients with viral-induced chronic liver disease, we investigated the diagnostic accuracy of serum LC-SPIK in comparison and in combination with standard serologic biomarkers (i.e., alpha-fetoprotein, AFP; and protein induced by vitamin K absence or antagonist-II, PIVKA-II) [11] for the detection of HCC in patients with non-alcoholic fatty liver disease (NAFLD) cirrhosis.

## 2. Materials and Methods

### 2.1. Patients

This single-center, retrospective, case–control study included patients with NAFLD cirrhosis that underwent US screening for hepatic nodular lesions at the outpatient clinic of the Unit of Gastroenterology, A.O.U. Città della Salute e della Scienza di Torino—Molinette Hospital between November 2012 and April 2021.

For inclusion in the study, patients fulfilled all the following criteria: age ≥ 18 years, diagnosis of NAFLD due to cirrhosis, availability of a serum sample collected at the time of US, and provision of written informed consent. Exclusion criteria were a history or presence of other concomitant liver diseases, ethanol consumption > 140 g/week for women and >210 g/week for men, and treatment with vitamin K antagonists.

The diagnosis of NAFLD as an etiologic factor of liver cirrhosis was made by liver biopsy showing histological stigmata of non-alcoholic steatohepatitis (NASH) [12]. In case of unavailability of liver biopsy or inconclusive results at histology, the assessment of NAFLD was made by excluding any other causes of liver disease, together with the presence of features of metabolic syndrome, namely body mass index (BMI) ≥ 30 kg/m^2^ and/or type 2 diabetes mellitus (T2DM), or at least two of the following: waist circumference ≥ 102/88 (males/females) cm, arterial hypertension, hypertriglyceridemia, low high-density lipoprotein (HDL) cholesterol [13].

The diagnosis of cirrhosis was achieved by histologic examination [12], or by vibration-controlled transient elastography and controlled attenuation parameter (FibroScan^®^, Echosens™, Paris, France) [14], or by hepatic US features and endoscopic signs of portal hypertension [15,16]. The diagnosis of HCC was mainly achieved by contrast-enhanced magnetic resonance imaging or computed tomography; in case of inconclusive imaging diagnosis, a biopsy of the suspected lesion was performed for pathological assessment [4]. HCC was classified according to the modified Barcelona Clinic Liver Cancer (BCLC) staging system [17]. 

### 2.2. Circulating Biomarkers

Serum samples collected in polypropylene 2 mL tubes, labeled with the study participant identification code, and stored at −80 °C were used for the measurement of circulating biomarkers. Personnel performing the analysis were blinded to the clinical characteristics of the patients included in the study. The serum levels of AFP and PIVKA-II were measured by chemiluminescence immunoassay on the LUMIPULSE G600 II platform (Fujirebio Inc., Tokyo, Japan) using Lumipulse^®^ G AFP-N (assay precision <3%) and Lumipulse^®^ G PIVKA-II (assay precision <4.4%), respectively [18]. The method was a two-step sandwich immunoassay, based on microparticle technology; microparticles were coated with monoclonal antibodies directed towards the antigens (i.e., AFP or PIVKA-II, respectively) present in serum samples, leading to the formation of antigen–antibody complexes. A secondary monoclonal antibody labeled with alkaline phosphatase (conjugate) recognized the immunocomplexes, leading to the formation of additional immunocomplexes. The subsequent addition of the substrate solution resulted in a chemiluminescent reaction, which was measured as relative light units; then, the results were automatically calculated according to a previously established calibration curve. The lower limit of detection was 0.075 ng/mL for AFP, 1.37 mAU/mL for PIVKA-II. 

Serum LC-SPIK values were determined by enzyme-linked immunosorbent assay (ImCare Biotech, Doylestown, PA, USA) as described in detail by Lu and colleagues [10].

### 2.3. Statistical Analysis

Categorical variables were reported as a number (*n*) and percentage; comparison between groups was performed by Fishers’ exact test or chi-squared (χ^2^) test, where appropriate. Quantitative continuous variables were reported as median and interquartile ranges (IQR); the D’Agostino–Pearson test was used to check for normal distribution. Continuous variables were compared by Mann–Whitney or Kruskal–Wallis test. The correlation between continuous variables was assessed by Spearman’s rank correlation coefficient (*r_s_*). The performance for HCC detection of serum LC-SPIK, AFP, and PIVKA-II was assessed by receiver operating characteristic (ROC) curve analysis and reported as the area under the curve (AUC). Cut-offs maximizing sensitivity (Se) and specificity (Sp) were calculated by Youden’s J statistic. The DeLong test was used to compare the performance of biomarkers. Multivariate logistic regression analysis was performed to evaluate the strength of association (reported as odds ratio (OR) and 95% confidence interval (CI)) of each single biomarker with the diagnosis of HCC, while adjusting for potential confounding factors, and to combine serum biomarkers into a model for HCC prediction. The same analysis was repeated using a 5-fold cross-validation approach (shuffling samples 20 times) to assess the performance of the model as previously described [14,19].

We considered a two-tailed *p* < 0.05 (two-tailed) statistically significant. Standard statistical analyses were performed using MedCalc software, v.18.9.1 (MedCalc bvba, Ostend, Belgium) while cross-validation was performed through the scikit-learn package, v.0.24.2, in the Python environment.

## 3. Results

### 3.1. Patients’ Characteristics

One-hundred-twenty patients with NAFLD cirrhosis were included in the study (Table 1). The median age was 65 (60–70) years; most patients were males (*n* = 78; 65%). Concerning the demographic characteristics, patients with HCC (*n* = 62) were slightly older than patients without tumor (*n* = 58) (66, 62–70 years vs. 63, 57–69; *p* = 0.016); the prevalence of males was higher in patients with HCC compared to those with cirrhosis (*p* < 0.001). We observed no differences in T2DM (*p* = 0.257), dyslipidemia (*p* = 0.455), and arterial hypertension (*p* = 0.691); despite a trend towards different BMI values, no differences were observed in the prevalence of obesity between patients with cirrhosis and those with HCC (*p* = 0.278). 

Patients with HCC had a more compromised liver function as compared to patients without tumor, showing lower rates of Child–Pugh score A (74% vs. 98%; *p* < 0.001). Consistently, the former showed lower platelet count (113 × 10^9^/L vs. 183 × 10^9^/L; *p* < 0.001), lower serum albumin values (3.5 g/dL vs. 4.2 g/dL; *p* < 0.001), higher total bilirubin levels (1.2 mg/dL vs. 0.7 mg/dL; *p* = 0.001), and higher INR values (1.25 vs. 1.09; *p* < 0.001) as compared to the latter. Among patients with HCC, 38 (61%) had a diagnosis of early-stage tumor (BCLC 0/A).

### 3.2. Circulating Biomarkers Values

**Figure 1:**
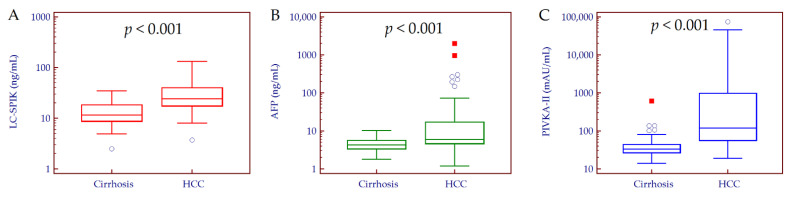
LC-SPIK (**A**), AFP (**B**), and PIVKA-II (**C**) median values in patients with and without HCC. Comparison between patients with cirrhosis and those with HCC was performed by Mann–Whitney test. Biomarkers’ values are depicted in Log scale. Patients with cirrhosis, *n* = 58; patients with HCC, *n* = 62. Abbreviations: alpha-fetoprotein (AFP), hepatocellular carcinoma (HCC), liver cancer-specific isoform of serine protease inhibitor Kazal (LC-SPIK), induced by vitamin K absence or antagonist-II (PIVKA-II).

The median serum levels of LC-SPIK, AFP, and PIVKA-II were significantly higher in patients with HCC compared to those without tumor (LC-SPIK: 24.3, 17.6–39.8 ng/mL vs. 11.7, 8.7–18.2 ng/mL; *p* < 0.001. AFP: 6.0, 4.6–16.9 ng/mL vs. 4.3, 3.4–5.6 ng/mL; *p* < 0.001. PIVKA-II: 121, 57–990 mAU/mL vs. 33, 27–44 mAU/mL; *p* < 0.001) (Figure 1). To note, 23 out of 62 patients with HCC (37%) showed false-negative AFP results.

Overall, the LC-SPIK serum values were correlated with age (*r_s_* = 0.34, *p* < 0.001), albumin (*r_s_* = −0.40, *p* < 0.001), INR (*r_s_* = 0.24, *p* < 0.001), and PIVKA-II (*r_s_* = 0.37, *p* < 0.001), while no significant correlation was observed with ALT (*r_s_* = −0.12, *p* = 0.193), AST (*r_s_* = 0.03, *p* = 0.763), platelets (*r_s_* = −0.12, *p* = 0.214), total bilirubin (*r_s_* = 0.04, *p* = 0.693), and AFP (*r_s_* = 0.11, *p* = 0.232). In patients with HCC, we observed a stepwise increase in the serum LC-SPIK values in advanced HCC stages (BCLC C/D) as compared to early and intermediate stages (BCLC 0/A/B) (43.4, 34.5–64.2 ng/mL vs. 23.2, 16.2–37.2 ng/mL, respectively; *p* = 0.003).

### 3.3. Biomarkers Performance for HCC Detection

**Figure 2:**
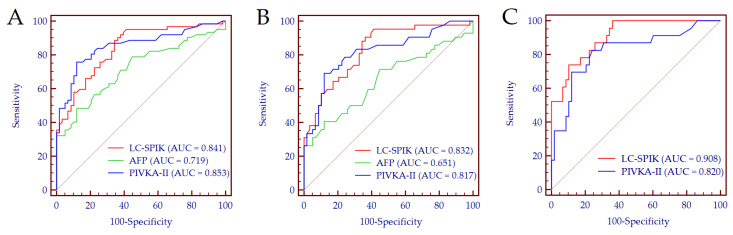
Biomarkers performance for the detection of HCC in the whole cohort (**A**), for the detection of early-HCC (**B**), and for the detection of HCC in patients with false-negative AFP results (**C**). (**A**): Patients with cirrhosis, *n* = 58; patients with HCC, *n* = 62. (**B**): Patients with cirrhosis, *n* = 58; patients with early-stage HCC, *n* = 38. (**C**): Patients with cirrhosis, *n* = 58; patients with AFP-negative-HCC, *n* = 23. Abbreviations: alpha-fetoprotein (AFP), area under the curve (AUC), liver cancer-specific isoform of serine protease inhibitor Kazal (LC-SPIK), induced by vitamin K absence or antagonist-II (PIVKA-II).

The diagnostic accuracy of LC-SPIK, AFP, and PIVKA-II for the detection of HCC was 0.841 (95% CI 0.763–0.901), 0.719 (95% CI 0.630–0.797), and 0.853 (95% CI 0.777–0.911), respectively (Figure 2A); no significant difference was observed between the performance of LC-SPIK and PIVKA-II (DeLong test: *p* = 0.809). Using 15.0 ng/mL as a cut-off value for serum LC-SPIK (Youden index), the Se and Sp for HCC detection were 89% and 66%, respectively. Notably, the performance of LC-SPIK did not diminish in detecting early-stage HCC (AUC = 0.832, 95% CI 0.744–0.899) (Figure 2B), while it increased in patients with false-negative AFP results (AUC = 0.908, 95% CI 0.823–0.961) (Figure 2C). 

To control for potential confounding factors, we analyzed the association between LC-SPIK and HCC diagnosis by a multivariate regression analysis adjusted for age, gender, Child–Pugh score, and triglycerides; remarkably, LC-SPIK > 15.0 ng/mL was significantly and independently associated with HCC (adjusted OR = 7.00, 95% CI 2.37–20.68; *p* < 0.001). 

**Figure 3:**
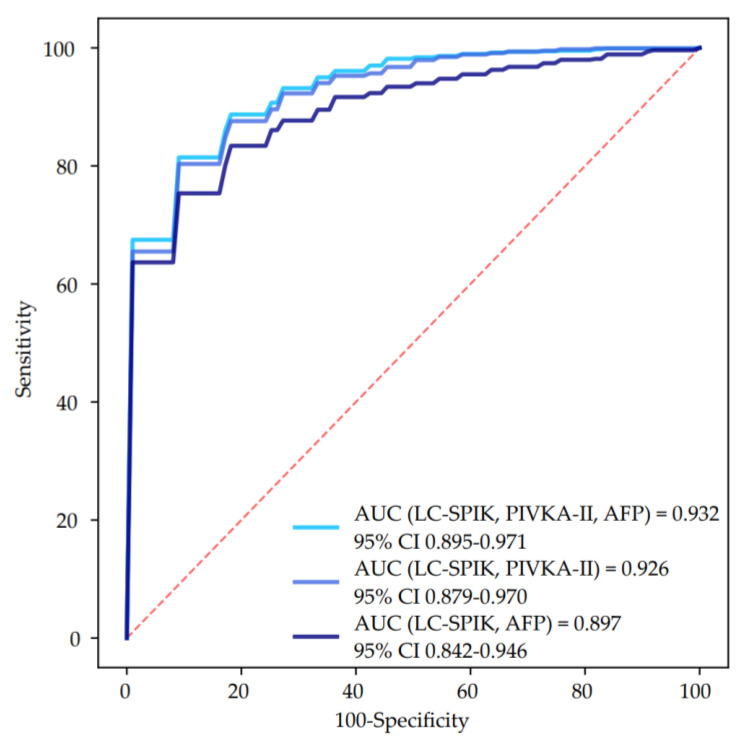
ROC curves of the combination of LC-SPIK with AFP and/or PIVKA-II for the detection of HCC. Abbreviations: alpha-fetoprotein (AFP), area under the curve (AUC), confidence interval (CI), liver cancer-specific isoform of serine protease inhibitor Kazal (LC-SPIK), protein induced by vitamin K absence or antagonist II (PIVKA-II).

Finally, we tested whether the combination of LC-SPIK with AFP and/or PIVKA-II could further improve the diagnostic accuracy for HCC detection. The higher accuracy was observed for the combination of LC-SPIK + AFP + PIVKA-II (AUC = 0.932, 95% CI 0.895–0.971), followed by LC-SPIK + PIVKA-II (AUC = 0.926, 95% CI 0.879–0.970), and LC-SPIK + AFP (AUC = 0.897, 95% CI 0.842–0.946) (Figure 3), while the combination of the three biomarkers was significantly superior to the combination of LC-SPIK + AFP (DeLong test: *p* = 0.036), no difference was observed when compared to the combination of LC-SPIK + PIVKA-II (DeLong test: *p* = 0.317).

## 4. Discussion

In the present study, we observed a good diagnostic accuracy of serum LC-SPIK for the detection of HCC in patients with NAFLD cirrhosis; the biomarker exhibited considerable performance even in early-stage HCC and in patients with false-negative AFP results. Furthermore, the combined use of serum LC-SPIK and PIVKA-II further increased the accuracy of HCC detection. These results provide additional evidence regarding the diagnostic value of LC-SPIK not only in patients with cirrhosis and HCC of viral etiology [10] but also in patients with dysmetabolic liver disease.

Chronic liver diseases due to metabolic disorders are common conditions with a rising burden worldwide, paralleling the epidemic of obesity and T2DM [20]. Among all causes of chronic liver disease, in our region, we observed that chronic hepatitis C virus infection significantly decreased from 41% in 1998 to 31% in 2014, while liver diseases due to metabolic disorders increased from 31% to 41% in the same period [21]. Consistently, the new diagnoses of HCC in patients with dysmetabolic liver disease progressively increased from 2011 (6.7%) to 2021 (40.3%), while HCCs developed on a background of advanced liver disease of viral etiology distinctly decreased [22]. 

In this setting, an additional caveat regarding the onset of HCC in patients with dysmetabolic liver disease is represented by the possibility of tumor occurrence even in the absence of cirrhosis (up to 30% of NAFLD-related HCCs) [23]; furthermore, the low disease awareness leads to diagnostics delays contributing to advanced-stage diagnosis of HCC and thus to reduced survival [24]. Therefore, the availability of effective tools able to improve the surveillance of patients with dysmetabolic liver disorders at risk of HCC development is highly desirable. In this regard, the accuracy demonstrated by LC-SPIK in our study cohort is promising, especially for the detection of early-stage and AFP-negative HCCs. Moreover, we observed that the combined used of LC-SPIK + PIVKA-II further improved the performance for HCC detection. While it has been shown by several recent studies that PIVKA-II had great potential for the prediction of HCC development in patients at risk [25,26,27,28], no data are available on the predictiveness of LC-SPIK. In this regard, future studies are needed to elucidate the ability of LC-SPIK, alone or in combination with PIVKA-II, to stratify the risk of HCC occurrence in patients under surveillance.

The results of the present study could be limited by the small number of patients enrolled, the heterogeneity of patients’ population, and by the lack of a validation cohort. The low number of patients analyzed did not allow us to perform any stratification concerning age, gender, etc.; however, we performed an adjusted multivariate regression analysis corrected for age, gender, Child–Pugh score, and triglycerides, and we observed that LC-SPIK values were significantly and independently associated to HCC. Another important limitation is the heterogeneity of the patients included, in particular those with HCC. To partially overcome this issue, we performed specific sub-analyses to investigate the accuracy of the biomarkers for the detection of early-stage HCC and AFP-negative HCC; in both cases, LC-SPIK still exhibited a good discriminatory ability. Despite our preliminary results, our results are in agreement with those published by Lu and colleagues in a cohort of patients with viral-related chronic liver disease [10]. Furthermore, we included in our study only patients with cirrhosis, who are at higher risk of HCC development; despite setting more stringent conditions for the evaluation of the biomarkers’ performance, the results still showed high accuracy for LC-SPIK in discriminating patients with HCC from those without tumor. Finally, we applied an artificial-intelligence approach based on a machine learning stratified cross-validation analysis to limit the potential bias derived from the lack of a validation cohort; this method reduces the risk of overfitting and provides the most accurate estimate of out-of-sample accuracy.

## 5. Conclusions

In conclusion, we provided preliminary evidence of the good diagnostic accuracy of the measurement of serum LC-SPIK in patients with NAFLD cirrhosis for the detection of HCC resembling the results previously observed in patients with chronic liver disease of viral etiology.

## Figures and Tables

**Table 1:** Demographic, biochemical, and clinical characteristics of the study population.

Variables	Cirrhosis	HCC	*p* Value
Patients, *n*	58	62	
Age (years), median (IQR)	63 (57–69)	66 (62–70)	0.016
Gender (M/F)	29/29	49/13	0.001
BMI (kg/m^2^), median (IQR)	31.0 (27.1–34.0)	29.8 (26.0–32.0)	0.043
Obesity (BMI ≥ 30.0 kg/m^2^), *n* (%)	34 (59%)	30 (48%)	0.278
T2DM, *n* (%)	40 (69%)	36 (58%)	0.257
Dyslipidemia, *n* (%) *	38 (66%)	36 (58%)	0.455
Arterial hypertension, *n* (%)	42 (72%)	42 (68%)	0.691
Child-Pugh score, *n* (%)			<0.001
A	57 (98%)	46 (74%)	
B	1 (2%)	13 (21%)	
C	0	3 (5%)	
ALT (U/L), median (IQR)	41 (25–64)	34 (25–45)	0.056
AST (U/L), median (IQR)	43 (31–55)	39 (33–56)	0.727
Platelets (×10^9^/L)	183 (138–223)	113 (81–151)	<0.001
Albumin (g/dL), median (IQR)	4.2 (4.0–4.4)	3.5 (3.0–4.3)	<0.001
Total bilirubin (mg/dL), median (IQR)	0.7 (0.5–1.1)	1.2 (0.7–1.7)	0.001
INR, median (IQR)	1.09 (1.04–1.16)	1.25 (1.11–1.41)	<0.001
Fasting glucose (mg/dL), median (IQR)	116 (97–139)	120 (95–143)	0.936
Total cholesterol (mg/dL), median (IQR)	177 (153–202)	164 (131–185)	0.078
HDL-cholesterol (mg/dL), median (IQR)	47 (39–63)	45 (35–54)	0.290
Triglycerides (mg/dL), median (IQR)	127 (101–175)	104 (79–136)	0.009
BCLC staging, *n* (%)			
0		7 (11%)	
A		31 (50%)	
B		14 (23%)	
C		7 (11%)	
D		3 (5%)	
HCC nodules, *n* (%)			
1		28 (45%)	
2		12 (19%)	
3		8 (13%)	
>3		14 (23%)	
Size of major nodule (mm), median (IQR)		27 (20–42)	

* Dyslipidemia was defined according to the presence of total cholesterol values ≥ 200 mg/dL and/or HDL-cholesterol values < 40 mg/dL for men and <50 mg/dL for women and/or triglycerides values ≥ 150 mg/dL. Abbreviations: alanine aminotransferase (ALT), aspartate aminotransferase (AST), Barcelona Clinic Liver Cancer (BCLC), body mass index (BMI), female (F), hepatocellular carcinoma (HCC), high-density lipoprotein (HDL), international normalized ratio (INR), interquartile range (IQR), male (M), number (*n*), type 2 diabetes mellitus (T2DM).

## Data Availability

The data presented in this study are available on request from the corresponding author.
